# Genome analysis of *Campylobacter jejuni* strains isolated from a waterborne outbreak

**DOI:** 10.1186/1471-2164-15-768

**Published:** 2014-09-08

**Authors:** Joana Revez, Ann-Katrin Llarena, Thomas Schott, Markku Kuusi, Marjaana Hakkinen, Rauni Kivistö, Marja-Liisa Hänninen, Mirko Rossi

**Affiliations:** Department of Food Hygiene and Environmental Health, Faculty of Veterinary Medicine, University of Helsinki, P.O. Box 66, (Agnes Sjöberginkatu 2), Helsinki, FI-00014 Finland; Biology Oceanography, Leibniz Institute for Baltic Sea Research, Rostock-Warnemünde, Germany; National Institute for Health and Welfare, Helsinki, Finland; Research Department, Finnish Food Safety Authority, Helsinki, Finland

**Keywords:** Waterborne outbreak, Campylobacteriosis, *Campylobacter jejuni*, Whole genome sequencing, PFGE, SNP, Phage, Integrated element, Microevolution

## Abstract

**Background:**

Waterborne *Campylobacter jejuni* outbreaks are common in the Nordic countries, and PFGE (pulsed field gel electrophoresis) remains the genotyping method of choice in outbreak investigations. However, PFGE cannot assess the clonal relationship between isolates, leading to difficulties in molecular epidemiological investigations. Here, we explored the applicability of whole genome sequencing to outbreak investigation by re-analysing three *C. jejuni* strains (one isolated from water and two from patients) from an earlier resolved Finnish waterborne outbreak from the year 2000.

**Results:**

One of the patient strains had the same PFGE profile, as well as an identical overall gene synteny and three polymorphisms in comparison with the water strain. However, the other patient isolate, which showed only minor differences in the PFGE pattern relative to the water strain, harboured several polymorphisms as well as rearrangements in the integrated element CJIE2. We reconstructed the genealogy of these strains with ClonalFrame including in the analysis four *C. jejuni* isolated from chicken in 2012 having the same PFGE profile and sequence type as the outbreak strains. The three outbreak strains exhibited a paraphyletic relationship, implying that the drinking water from 2000 was probably contaminated with at least two different, but related, *C. jejuni* strains.

**Conclusions:**

Our results emphasize the capability of whole genome sequencing to unambiguously resolve the clonal relationship between isolates of *C. jejuni* in an outbreak situation and evaluate the diversity of the *C. jejuni* population.

**Electronic supplementary material:**

The online version of this article (doi:10.1186/1471-2164-15-768) contains supplementary material, which is available to authorized users.

## Background

*Campylobacter* spp. are recognized as the leading cause of human bacterial gastroenteritis in the industrialized world [1]. In the European Union (EU), the incidence of human campylobacteriosis cases has followed an increasing trend in recent times and it continues to be the most commonly reported zoonosis with 214,268 confirmed cases in 2012 [2]. The majority of the infections are sporadic and seasonal, with a clear incidence peak in the summer months and early autumn [1]. Although infrequently reported compared with sporadic cases, outbreaks of campylobacteriosis do occur and are often associated with the consumption of raw milk and contaminated drinking water [1–3]. In Finland, waterborne outbreaks caused by enteric pathogens are commonly registered [4–7] and *C. jejuni* was the causative agent in 19% of the recorded outbreaks between 1998 and 2011 (http://www.thl.fi). This corresponds to approximately two *C. jejuni* waterborne outbreaks annually. Resolving *C. jejuni* outbreaks is complicated due to a prolonged lag time. A long incubation period (from 2 to 7 days) and lengthy diagnostic procedures cause an estimated lag time of approximately 2 weeks between time of exposure and recognition of the waterborne transmission [8]. This lag time may hinder the ability to detect *C. jejuni* from the water source, especially if the drinking water was transiently contaminated [8, 9].

PFGE (pulsed-field gel electrophoresis) typing of isolates has been widely used in outbreak investigations. PFGE is considered to be the gold standard for source tracking [8] due to the reported stability of PFGE genotypes in different host populations (e.g. human and chicken), irrespective of temporal and geographical space [10–12]. However, PFGE profiles cannot conclusively establish the clonal relationship between isolates, affecting the epidemiological investigations. Bacterial strains with identical PFGE or highly similar profiles isolated years apart generally show genetic diversity accumulated by genetic drift, homologous recombination or horizontal gene transfer [10, 13]. On the contrary, the genomic differences between epidemiologically linked isolates sharing PFGE profiles are expected to be minor since the strains are considered to be the recent expansion of a single clone [14]. However, due to limited resolution capacity similar PFGE profiles could overestimate the clonal relationship between isolates [15, 16]. Furthermore, since alterations in the PFGE patterns can result from a single genetic event due to a single-nucleotide polymorphism in a restriction site [9], bacteriophage acquisition or loss or transposition [17, 18], a clonal relationship may exist even between strains with different PFGE profiles [9].

Whole-genome sequencing (WGS) has recently been utilized to increase resolution power in the analysis of outbreak-associated isolates, leading to faster and more precise source identification in outbreak investigations, and to discriminate between alternative epidemiological hypotheses [16, 19]. The aim of this study was to explore the applicability of WGS to an outbreak investigation by comparing the genomes of *C. jejuni* isolates from a Finnish waterborne outbreak that had occurred in 2000. The outbreak had already been resolved using both epidemiological and environmental analysis tools. All isolates have Penner serotype 12 and their *Kpn*I and *Sac*II profiles were identical, except for one patient isolate that had a three-band difference in the *Kpn*I profile and a two-band difference in the *Sac*II profile [4].

## Results and discussion

### Genome of *C. jejuni*water isolate 4031 and identification of mobile genetic elements

The combination of paired-end and 5 kb mate-pair library allowed the complete assembly of the genome of *C. jejuni* strain 4031, consisting of a single chromosome of 1,669,329 nucleotides. Plasmid DNA was not detected. A total of 1,697 coding DNA sequences (CDSs) were identified in a coding area of 94.28% and function was predicted for ~73%. The strain belongs to ST-45 and, as previously observed in certain strains of the ST-45 complex, gamma-glutamyl transpeptidase, fucose permease and a secreted L-asparaginase were not detected [20]. The lipooligosaccharide (LOS) locus resembles class P of *C. jejuni* GB4 [21, 22], which is associated with a non-sialylated LOS outer core structure without ganglioside mimicry [22]. IslandViewer predicted the presence of a putative prophage of 36,567 bp (from 441,523 bp to 478,090 bp) integrated between locus BN867_04520 (translation elongation factor G, homologue to Cj0493 of *C. jejuni* NCTC 11168) and locus BN867_05040 (hypothetical protein, homologue to Cj0494). This prophage showed 85.6% global nucleotide identity with the integrated element CJIE2 identified in *C. jejuni* RM1221 (calculated using Needleman-Wunsch global alignment algorithm) and it is integrated in the same region of the chromosome. The CJIE2 element in *C. jejuni* 4031 includes 51 open reading frames (ORFs), but a putative function was predicted for only three of these (BN867_04720, endonuclease; BN867_04740, phage repressor protein; BN867_04810, terminase B protein). Upon manual inspection, a second region that probably also has a phage origin was detected to be inserted between nucleotide 665,673 (corresponding to locus BN867_06900) and 670,480 (corresponding to locus BN867_06990). Pairwise comparison with *C. jejuni* RM1221 revealed that this region corresponds to a vestigial Mu-like phage of approximately 4,807 bp (CJIE1; CJE0213-CJE0275): BN867_06900 is a homologue to CJE0275, and BN867_06990 is a homologue to CJE0213. The vestigial Mu-like phage of *C. jejuni* 4031 is integrated in a different region than in *C. jejuni* RM1221: it is located upstream to the invasion phenotypic protein (BN867_0700/BN867_0710; *cipA*). A vestigial Mu-like phage integrated upstream of *cipA* is also present in the genome of *C. jejuni* M1 (ST-137, ST-45 complex).

### The human outbreak-associated isolate IHV116292 underwent genome rearrangement

The human *C. jejuni* isolates IHV116260 and IHV116292 were sequenced using paired-end library, assembled and mapped against *C. jejuni* 4031.

The *Kpn*I PFGE profile of the human isolate IHV116292 differed from that of *C. jejuni* 4031 and IHV116060 by three bands [4], which were interpreted to mean that the isolate was closely related to the outbreak strain [23]. However, the assembled contigs of IHV116292 did not map unequivocally to the genome of *C. jejuni* 4031. On the contrary, all contigs of *C. jejuni* IHV116260 mapped completely to the water isolate genome.

**Figure 1:**
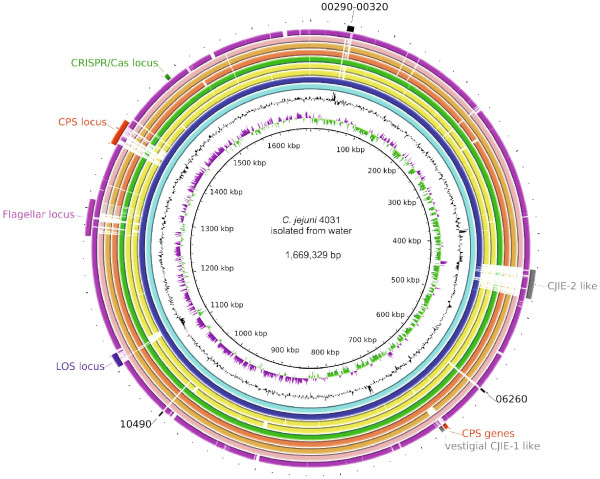
**BLAST atlas produced using BLAST Ring Image Generator v 0.95.** In the middle, a genome atlas of *C. jejuni* 4031 strain is shown, around which BLAST lanes are shown. Every lane corresponds to a genome. From in to out: GC skew; GC content; BLASTN pairwise comparison of *C. jejuni* genomes: IHV116260 (human waterborne outbreak); IHV116292 (human waterborne outbreak); BIGS0004, ST-45 (chicken); 55037, ST-45 (chicken); 4028, ST-1971 (chicken farm environment); M1, ST-137 (human); 327, ST-230 (turkey); 81116, ST-267 (human); RM1221, ST-354 (human); annotation: green, CRISPR/Cas locus; red, CPS locus/genes; purple, Flagellar locus; blue, LOS locus; grey, *Campylobacter* integrate elements; black, locus_tags of *C. jejuni* 4031 (BN867_).

Initial comparison between the outbreak isolates as well as other available genomes of *C. jejuni* strains belonging to the ST-45 was performed using BLASTN with default parameters and an atlas was built using *C. jejuni* 4031 as the reference genome (Figure 1). The more divergent genomic regions of all analysed ST-45 complex genomes (4031, IHV116260, IHV116292, BIGS0004, 55037, 4028, M1, 327) included the LOS, flagella and capsule (CPS) loci, as well as CJIE2 which was only present in the three outbreak isolates. As expected, the genomes of human isolates were highly similar to the reference genome of the water isolate. However, significant variation was detected within the CJIE2 region between the isolates IHV116292 and 4031.

**Figure 2:**
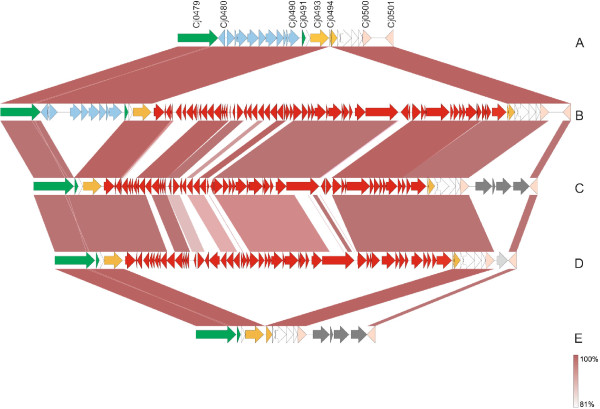
**A collinear BLASTN comparison of the CJIE2 elements and the up- and downstream adjacent regions of** ***C. jejuni*** **strains (B) RM1221, (C) IHV116292, (D) 4031 with the corresponding region in** ***C. jejuni*** **strain (A) NCTC 11168 and (E) M1.** Colour scheme: red, CJIE2; blue, fucose locus; dark grey, Cj0967-Cj0975-homolog cluster; green, conserved genes up- and downstream the fucose cluster; orange, conserved genes up- and downstream the CJIE2; pink, conserved genes up- and downstream the Cj0967-Cj0975-homolog cluster; grey, ammonium transporter.

**Figure 3:**
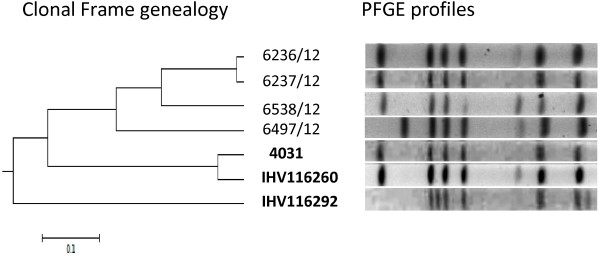
**ClonalFrame genealogy and PFGE profiles of** ***C. jejuni*** **strain 4031, IHV116260, IHV116292, 6236/12, 6237/12, 6538/12 and 6497/12.**

A collinear BLASTN comparison of the CJIE2 elements and the up- and downstream adjacent regions is shown in Figure 2. The CJIE2 of IHV116292 is 37,058 bp in length, includes 49 ORFs and shows 84.3% global nucleotide identity with CJIE2 of *C. jejuni* 4031 (calculated using Needleman-Wunsch global alignment algorithm) with the most divergent part located in the central 22 kb region. In addition, the CJIE2 of the genome of *C. jejuni* IHV116292 possesses a *Kpn*I restriction site (in the gene immediately upstream of the Cj0594 homologue) which is not present in the CJIE2 of *C. jejuni* 4031. This additional restriction site explains the differences detected in *Kpn*I patterns of IHV116292 (Figure 3).

*C. jejuni* 4031 and IHV116260 differ also from IHV116292 by an insertion of approximately 6 kb downstream of CJIE2, located between tRNA-2-selenouride synthase (*ybbB*) and ferrochetolase (*hemH*). A second copy of this locus occurs downstream the Acyl-CoA thioester hydrolase (Cj0965c of *C. jejuni* NCTC 11168) and it is a homologue to the locus Cj0967-Cj0975 of *C. jejuni* NCTC 11168. The Cj0967-Cj0975 locus downstream of Cj0965c, as observed in IHV116292, is conserved among several *C. jejuni* strains including ST-45. By contrast, the second copy of this locus inserted between *ybbB* and *hemH* has been previously described only in *C. jejuni* 81–176 [24] and BLASTN analysis showed that it is present also in *C. jejuni* M1 (Figure 2), 81116 and ICDCCJ07001 (data not shown). As described for *C. jejuni* 81–176 [24], both these loci in *C. jejuni* 4031 harbour a ~200 bp specific region of intergenic AT-rich DNA, including an inverted repeat and a G-rich tract upstream the start codon of the Cj0967 homologue. In the *C. jejuni* isolate IHV116292 the locus downstream of *ybbB* is substituted by a gene encoding an ammonium transporter showing 92.2% nucleotide identity with the pseudogene Cj0501 in *C. jejuni* NCTC 11168 and high amino acid identity with several *C. jejuni* and *C. coli* ammonium transporters. This gene is not present in the genome of either *C. jejuni* 4031 or IHV116260.

The analysis revealed that the human isolate IHV116292 contained several genome rearrangements inside and immediately downstream the CJIE2 element compared to the water isolate, and these were responsible for the observed differences in PFGE profiles. These differences were verified by PCR, thus they were not a consequence of misassembly or sequencing error. However, based on these data it cannot be determined whether these alterations have evolved in IHV116292 during the infection in the patient, or were already present in the population that caused the outbreak.

### SNP analysis and genealogy reconstruction suggest that *C. jejuni*4031 and IHV116292 are two different strains

To understand the microevolution of the waterborne outbreak-associated isolates, we analysed the polymorphic sites detected using progressiveMauve aligner. The polymorphic sites were checked manually if they fulfilled our criteria and they were divided into isolated SNPs and CNPs (see Methods). SNPs are most likely caused by mutations, whereas CNPs are probably the result of homologous recombination [25]. The human IHV116260 strain showed only three SNPs compared with the water strain 4031 (T→C 143820, methyl-accepting chemotaxis signal transduction protein BN867_01350/BN867_01360; G→T, CJIE2 terminase B protein, BN867_04810; C→G, LOS locus BN867_11290). In contrast, even when excluding the previously recognized variable regions (CJIE2 region and the Cj0967-Cj0975 duplicated locus downstream of *ybbB*), the human strain IHV116292 differed from *C. jejuni* 4031 by 69 SNPs, which are spread across the chromosome (see Additional file 1: Table S1). Furthermore, IHV116292 showed the presence of 9 CNPs affecting a total of 8 genes (see Additional file 1: Table S2).

Only a few SNPs are expected to be produced during a single passage through the host, based on previous studies in a human patient [26] and animal models [27, 28]. The few differences observed between IHV116260 and 4031 can be a consequence of a single passage through the patient. By contrast, the much greater number of polymorphisms observed between IHV116292 and 4031 suggests that they were not generated during the outbreak.

To verify this hypothesis we attempted to reconstruct the genealogy of the three isolates. For this analysis we included the genomes of four additional ST-45 *C. jejuni* strains isolated from chicken 12 years after the outbreak. The chicken isolates had a *Kpn*I PFGE profile indistinguishable from *C. jejuni* 4031 (6538/12; 6237/12; 6236/12) or differentiated by two bands (6497/12) (Figure 3). Two chicken strains (6237/12; 6236/12) were obtained from different houses of the same farm, while the other two strains were obtained from two different farms two weeks later. It is expected that in the time frame of 12 years the isolates would accumulate several SNPs by genetic drift, allowing us to estimate the distance between *C. jejuni* 4031 and IHV116292, assuming that similar PFGE patterns originated from a common ancestor. The genealogy reconstructed using ClonalFrame based on core genome alignment obtained with progressiveMauve is presented in Figure 3. The tree shows a monophyletic relationship between *C. jejuni* 4031, the human isolate IHV116260 and the chicken strains. The *C. jejuni* human isolate IHV116292 is located in a separate branch that originates directly from the root. A BLASTN comparison of the genomes of *C. jejuni* 4031 and the chicken strains revealed that they are very similar (see Additional file 2: Figure S1). Differences between *C. jejuni* 4031 and the chicken strains were located in the CJIE2 (absent in strain 6497/12), the LOS locus and the flagellar locus. To estimate the genetic distance between the chicken and the outbreak strains, we compared the allelic profiles of 1,287 genes obtained from the PubMLST-*Campylobacter* database. Split decomposition (see Additional file 2: Figure S2) showed that the chicken strains are closer to *C. jejuni* 4031 and the human isolate IHV116260 (average distance of 0.0061) than IHV116292 (average distance of 0.0175). We further calculated the number of SNPs present between *C. jejuni* 4031 and the chicken strain 6236/12 using the same criteria applied for the outbreak strains. In particular, we excluded all regions that could be affected by homologous recombination (CNPs, CJIE2) in order to detect only those polymorphisms most likely acquired by mutation. From an original list of ~1000 polymorphisms extracted from progressive Mauve alignment of *C. jejuni* 4031 and 6236/12, only 64 SNPs fulfilled our criteria (see Additional file 1: Table S3). In fact, the majority of the polymorphisms were located within the CJIE2 or were classified as CNPs, indicating that homologous recombination explained the accumulation of genetic differences between *C. jejuni* 4031 and 6236/12.

These results suggest that the number of differences observed between *C. jejuni* 4031 and IHV116292 is too large for accumulation over the course of the outbreak. On the basis of the results, two hypotheses can be formulated: 1) the water was contaminated by a mixture of at least two related *C. jejuni* strains and 2) IHV116292 is not associated with the outbreak. Considering the findings of the epidemiological investigation [4], the first hypothesis appears to be the most plausible.

### Recombination is the probable origin of the observed differences in CJIE2

CJIEs are postulated to be hypervariable genomic regions that contribute to diversity of *C. jejuni* [29]. In particular, CJIE1 (the Mu-like phage) has been shown to form a family of prophages with both conserved and divergent sequence regions, and appears to be adapted to *C. jejuni* [30]. Our analysis of three outbreak isolates showed that CJIE2 is variable even between highly related *C. jejuni* strains (Figure 2). We extended the comparison to include CJIE2 sequences of chicken strains of the same ST-45. Two different CJIE2 sequences were detected: 6236/12 and 6237/12 have an identical CJIE2 sequence, whereas 6538/12 possesses a CJIE2 that differs from the CJIE2 of 6236/12 and 6237/12 at three positions (see Additional file 2: Figure S3). Comparing the chicken-associated CJIE2 sequences with those of the outbreak strains, we observed that they are more related to CJIE2 of *C. jejuni* IHV116292 than to 4031, in spite of the ancestral relationship between the later and the chicken strains. This observation suggests that CJIE2 undergoes extensive recombination and genetic rearrangement, comparable with that of the Mu-like phage CJIE1. Considering that the endonucleases encoded by these elements inhibit natural transformation of *C. jejuni* [31], their hypervariability might influence the microevolution of closely related *C. jejuni* strains.

## Conclusions

Outbreak strains are isolates that are both epidemiologically (e.g., by time, site and common source) and genetically related (i.e. have indistinguishable genotypes). Such isolates are presumed to be clonal [23]. However, in waterborne outbreaks, several varieties of pathogens (e.g. viruses, protozoa and bacteria) or a mixture of strains are sometimes detected in the water as well as in human samples as a result of waste water contamination [2]. In such cases several different outbreak-associated strains may be detected [8]. In the waterborne outbreak re-investigated in this study, two human isolates were attributed to the water contamination, based on serotype and PFGE data [4]. Although one of the human isolates had a slightly deviant PFGE pattern, this was not considered significant enough to exclude it from the outbreak, as a PFGE profile can change after only a single passage through the chicken host [32], by genomic rearrangement due to phage infection [17] or mobilization of temperate phages [18]. The PFGE pattern differences observed in IHV116292 could potentially have occurred during the passage though the human host. However, our comparative genomic analysis clearly reveals that this human isolate contains so many genomic alterations compared to the water strain, that it represents another *C. jejuni* strain. In this particular case, whole genome analysis was required to correctly define the clonal frame. This study highlights the capability of whole-genome sequencing to unambiguously resolve the relationship between the isolates of a *C. jejuni* outbreak. In the future, next-generation sequencing technologies will more intensively be applied as a tool for outbreak strain characterization, remarkable improving the reliability of epidemiological conclusions on the association between source and infected patients.

## Methods

### Bacterial strains, PFGE and DNA isolation

Two waterborne outbreak-associated *C. jejuni* isolates collected from two patients (IHV116292 and IHV116260) and one isolate from contaminated tap water (4031) were selected. All of the *C. jejuni* isolates were collected in August 2000 during a large outbreak of gastroenteritis that had occurred in a community in southern Finland [4, 8]. This study was part of the public health response to a waterborne outbreak. According to Finnish legislation, no ethical approval is needed for this type of response. In addition, four chicken strains (6538/12, 6237/12, 6236/12 and 6497/12) isolated during summer 2012 over the course of the national *Campylobacter* monitoring programme were included. The strains were selected on the basis of their PFGE profile similarity to the outbreak isolates and having the same Multi Locus Sequence Type (MLST). The *Kpn*I PFGE patterns for the strains were produced as previously described [8]. High quality genomic DNA was isolated with the Wizard Genomic DNA Purification Kit (Promega, Germany), according to the manufacturer’s instructions.

### Genome sequencing, assembly and annotation

Genome sequences were obtained using Illumina sequencing technology with 100 cycles paired-end reads. In addition, a 5 kb mate-paired end library was performed for the isolate 4031. Illumina reads were trimmed using the Condetri perl script [33] with default settings, with a minimum read length of 75 nucleotides. All reads were assembled separately using MIRA [34, 35] and ABySS [36]. The genomes of *C. jejuni* strains 4031, IHV116260 and IHV116292 were closed, and for the chicken strains virtual genomes were generated. For this purpose, the water isolate *C. jejuni* 4031 was used as a scaffold, and the contigs were re-ordered using Mauve [37]. Primary annotation of all strains was performed using Rapid Annotation using Subsystem Technology (RAST) [38], and later, the sequences were manually curated using Artemis [39]. Prophages were searched in the genomes using IslandViewer [40, 41]. Clusters of hypothetical genes, generally associated with genomic islands [41], were searched and manually inspected.

### Comparative genomics

Genomes 4031 (water sample from waterborne outbreak, this study), IHV116260 (human waterborne outbreak, this study), IHV116292 (human waterborne outbreak, this study), BIGS0004, ST-45 (chicken; NCBI ANGO), 55037, ST-45 (chicken; NCBI AIOH01), 4028, ST-1971 (chicken farm environment; ENA PRJEB6225), M1, ST-137 (human; NCBI NC_017280), 327, ST-230 (turkey; NCBI ADHM01), 81116, ST-267 (human; NCBI NC_009839), RM1221, ST-354 (human; NCBI NC_003912) were compared using BLAST and the atlases were generated using BLAST Ring Image Generator v 0.95 (BRIG; [42]). Synteny was evaluated using Mauve [43] and Artemis Comparative Tool (ACT; [44]). Linear comparison of integrated elements was performed using EasyFig v2.1 [45]. Assembled data were uploaded on usmirror1.pubmlst.org/campylobacter/ database and implemented with the Bacterial Isolate Genome Sequence Database (BIGS-DB) software [46] and allelic profiles for all common loci were retrieved. Allelic profiles of all isolates were compared using Splitstree4 [47]. Lists of polymorphisms were exported from pairwise analyses performed using Mauve and then curated manually. Polymorphisms were filtered to remove those likely due to assembly or alignment errors. Polymorphisms were filtered if i) detected in or immediately adjacent to the ribosomal operon, ii) the 50 nucleotides surrounding the polymorphism were not unique in the genome (analysed by BLASTN) or iii) detected in a homopolymeric run. Nucleotide polymorphisms were divided in isolated single nucleotide polymorphisms (SNPs) and clusters of nucleotide polymorphisms (CNPs). SNPs were defined as polymorphisms separated from the next nucleotide polymorphism by a difference greater than 200 bp on both sides. CNPs were defined as groups of at least two polymorphisms with a distance of less than 200 bp between two consecutive polymorphic sites, separated from the next sequence by a difference greater than 200 bp on both sides. The clonal genealogy of the strains based on the whole genome was estimated using a model-based approach to determine bacterial microevolution implemented in ClonalFrame [48]. Genomes were aligned using progressiveMauve [43] and collinear blocks bigger than 500 bp were filtered using the perl script stripSubsetLCBs available in the ClonalOrigin package [49]. Thus, ClonalFrame was run with 10,000 burn-in iterations followed by 10,000 data collection iterations. The consensus tree represents combined data from three independent runs, with 75% consensus required for inference of relatedness.

### Data deposition

The genome of *C. jejuni* 4031 was submitted to EMBL with accession number HG428754. The sequence reads of the other strains were submitted to EMBL under project number PRJEB4165 (ERP003426).

## Electronic supplementary material

Additional file 1: Table S1: List of single nucleotide polymorphisms (SNPs) detected in *C. jejuni* IHV116292 compared with the reference strain *C. jejuni* 4031; **Table S2.** List of cluster of nucleotide polymorphisms (CNPs) detected in *C. jejuni* IHV116292 compared with the reference strain *C. jejuni* 4031; **Table S3.** List of single nucleotide polymorphisms (SNPs) detected in *C. jejuni* 6236/12 compared with the reference strain *C. jejuni* 4031. (XLSX 377 KB)

Additional file 2: Figure S1: BLAST atlas produced using BLAST Ring Image Generator v 0.95 comparing *C. jejuni* 4031 strain versus *C. jejuni* HV116260, IHV116292, 6497/12, 6538/12, 6236/12, 6237/12; **Figure S2.** (A) Split decomposition and (B) Distance matrix of the allelic profiles of 1,287 genes obtained from PubMLST-*Campylobacter* database of *C. jejuni* strains 4031, HV116260, IHV116292, 6497/12, 6538/12, 6236/12 and 6237/12; **Figure S3.** A collinear BLASTN comparison of the CJIE2 elements of *C. jejuni* strains RM1221, 4031, IHV116292, 6538/12 and 6236/12. (PDF 286 KB)
