# Supplementary material for: Genome analysis of Campylobacter jejuni strains isolated from a waterborne outbreak
Source: BMC Genomics. 2014 Sep 8;15(1):768. doi: 10.1186/1471-2164-15-768 (PMC4168118; doi:10.1186/1471-2164-15-768)
Supplement: Supplementary file 2 — Additional file 2: Figure S1: BLAST atlas produced using BLAST Ring Image Generator v 0.95 comparing C. jejuni 4031 strain versus C. jejuni HV116260, IHV116292, 6497/12, 6538/12, 6236/12, 6237/12; Figure S2. (A) Split decomposition and (B) Distance matrix of the allelic profiles of 1,287 genes obtained from PubMLST-Campylobacter database of C. jejuni strains 4031, HV116260, IHV116292, 6497/12, 6538/12, 6236/12 and 6237/12; Figure S3. A collinear BLASTN comparison of the CJIE2 elements of C. jejuni strains RM1221, 4031, IHV116292, 6538/12 and 6236/12. (PDF 286 KB) [file 12864_2013_6451_MOESM2_ESM.pdf]

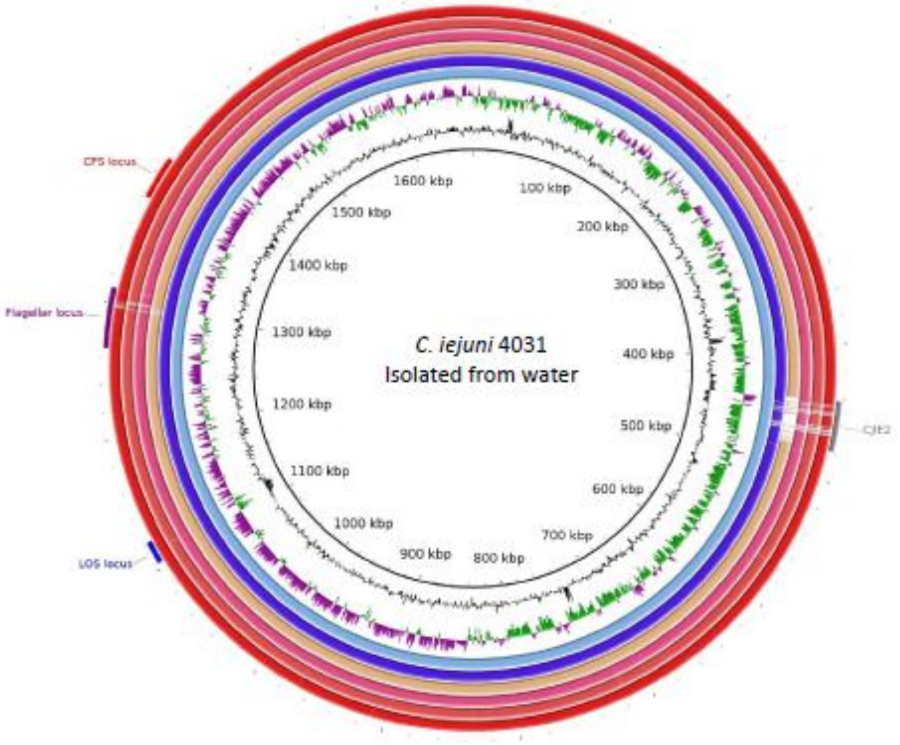

Additional file 2 Figure S1. BLAST atlas produced using BLAST Ring Image Generator v 0.95. In the middle, a genome atlas of *C. jejuni* 4031 strain is shown, around which BLAST lanes are shown. Every lane corresponds to a genome. From in to out: GC skew; GC content; BlastN pairwise comparison of *C. jejuni* genomes with ST-45: HV116260 (human waterborne outbreak); IHV116292 (human waterborne outbreak); 6497/12 (chicken); 6538/12 (chicken); 6236/12 (chicken); 6237/12 (chicken); annotation: red, CPS locus/genes; purple, Flagellar locus; blue, LOS locus; grey, *Campylobacter* integrate elements.

A

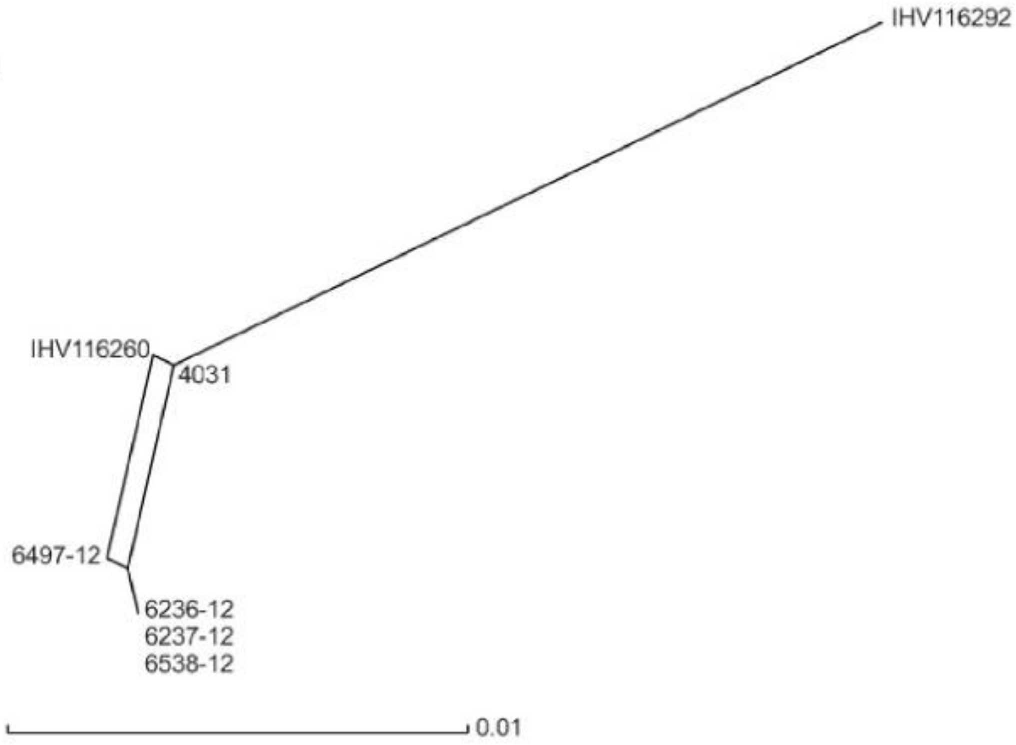

B

4031	0						
IHV116260	0.001						
IHV116292	0.018	0.017	0				
6236-12	0.007	0.006	0.023	0			
6237-12	0.007	0.006	0.023	0.000	0		
6497-12	0.005	0.005	0.023	0.002	0.002	0	
6538-12	0.007	0.006	0.023	0.000	0.000	0.002	0

Additional file 2: Figure S2. (A) Split decomposition and (B) Distance matrix of the allelic profiles of 1,287 genes obtained from PubMLST-*Campylobacter* database of *C. jejuni* strains 4031 (water), HV116260 (human waterborne outbreak), IHV116292 (human waterborne outbreak), 6497/12, ST-45 (chicken); 6538/12, ST-45 (chicken); 6236/12, ST-45 (chicken) and 6237/12, ST-45 (chicken).

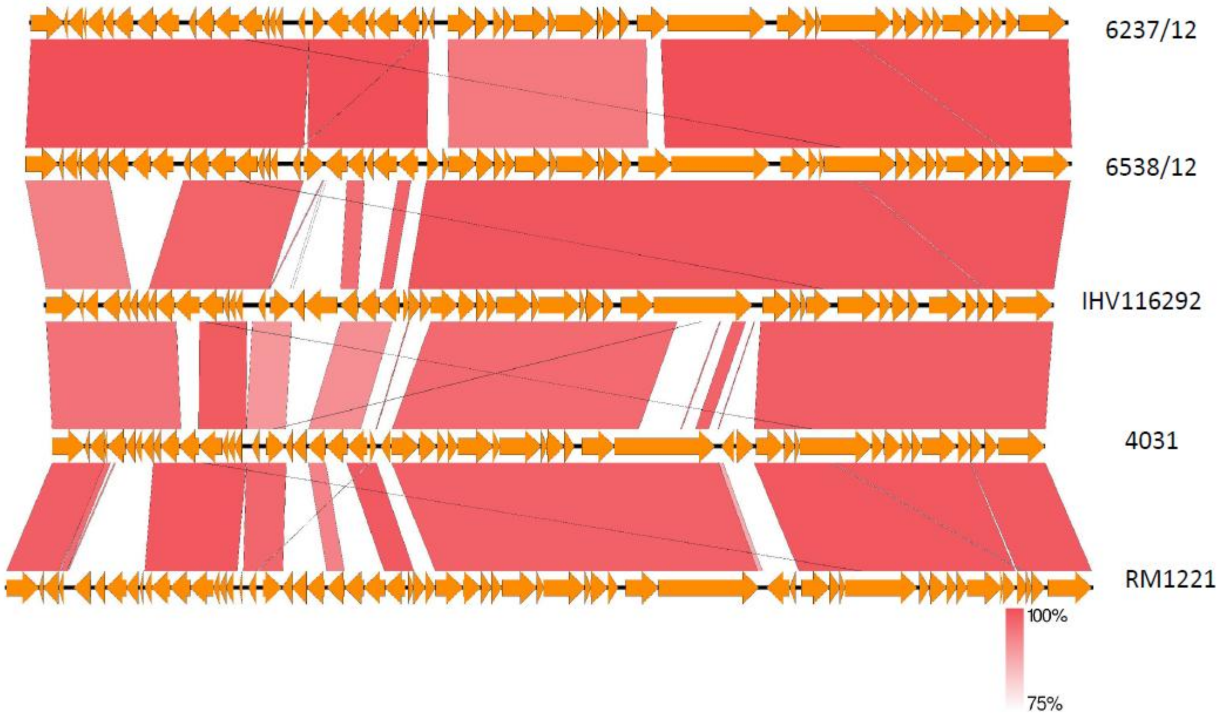

Additional file 2: Figure S3. A collinear BLASTN comparison of the CJIE2 elements of *C. jejuni* strains RM1221, 4031, IHV116292, 6538/12 and 6236/12.
